# Supplemental Dietary Selenohomolanthionine Improve Antioxidant Activity and Immune Function in Weaned Beagle Puppies

**DOI:** 10.3389/fvets.2021.728358

**Published:** 2021-09-30

**Authors:** Chunyan Shao, Moufeng Zheng, Ziwei Yu, Sheng Jiang, Bin Zhou, Quanjiang Song, Tianning Ma, Yingshan Zhou, Wanyu Dong, Ding Li, Yao Gu, Xiaodu Wang, Houhui Song

**Affiliations:** ^1^Key Laboratory of Applied Technology on Green-Eco-Healthy Animal Husbandry of Zhejiang Province, Zhejiang Provincial Engineering Laboratory for Animal Health Inspection and Internet Technology, College of Animal Science and Technology, College of Veterinary Medicine, Zhejiang A&F University, Hangzhou, China; ^2^ABNA Trading (Shanghai) Co., Ltd., Shanghai, China

**Keywords:** selenium, selenohomolanthionine, antioxidant activity, immune function, weaned puppies

## Abstract

The purpose of this study was to investigate the effects of dietary Selenohomolanthionine (SeHLan) on antioxidant status and immune response in canine parvovirus (CPV) vaccinated puppies. In this study, 30 weaned puppies were randomly divided into six groups: control group (–Se/–Vacc), immunization group (–Se/+Vacc), supplementation of sodium selenite group (SS/+Vacc, 0.35 mg/kg DM), low-dose SeHLan group (SeHLan-L/+Vacc, 0.35 mg/kg DM), mid-dose SeHLan group (SeHLan-M/+Vacc, 1.0 mg/kg DM), and high-dose SeHLan group (SeHLan-H/+Vacc, 2.0 mg/kg DM). The puppies were fed for 42 days and vaccinated with Vanguard Plus 5 on day 0 and day 21. Blood samples were collected on 7, 14, 21, 28, 35, 42 days post-immunization (PI) for determination of antioxidant indicators, lymphocyte proliferation index, serum cytokine concentration (IL-2, IL-4), canine polymorphonuclear neutrophils (PMN) phagocytic function, and the level of CPV antibody titers. The results showed that SeHLan supplementation raised the serum Se concentration and glutathione peroxidase (GSH-Px) activity in a dose-dependent manner (*P* < *0.05*). It also increased the activity of serum superoxide dismutase (SOD) and decreased serum malondialdehyde (MDA) content, especially in SeHLan-M/+Vacc group (1.0 mg/kg DM) (*P* < *0.01*). SeHLan supplementation significantly increased lymphocyte proliferation, IL-2, and IL-4 levels in canine serum, and enhanced phagocytosis of PMN in vaccinated puppies (*P* < *0.05*). Moreover, SeHLan supplementation shortened the CPV antibody production time and increased the CPV antibody titers (*P* < *0.05*). Of note, the beneficial effects of SeHLan were superior to those of SS. In conclusion, dietary SeHLan supplementation improved antioxidant activity, increased CPV antibody titers, and enhanced immune function in puppies after weaning. An appropriate dosage of SeHLan (1~2 mg/kg DM) may confer nutritional benefits in puppies.

## Introduction

Selenium (Se) is a trace element that is necessary for human and animal life activities and has also been used as a natural antioxidant (1–3). Besides, it is a cofactor of antioxidant enzymes such as glutathione peroxidase (GSH-Px), which is involved in the elimination of peroxides and hydroxyl free radicals produced during metabolism (4–7). Furthermore, studies have shown that Se plays an important role in regulating innate and adaptive immune activity (8, 9), such as improving pro-inflammatory gene expression in macrophages (10), enhancing neutrophils functions (11) and promoting Chlamydia antibody production in sheep (12). The immune-enhancing activity of Se is largely attributed to the scavenging of free radicals and neutralizing of reactive oxygen species (ROS), thus, reducing oxidative stress (13). Dietary Se supplements are mainly in two forms: inorganic Se and organic Se. At present, inorganic Se remains the main source of Se in poultry (14) and livestock (15) nutrition. However, the application of inorganic Se is reduced in humans and companion animals due to its high toxicity and low biological utilization rate (16).

Weaning is a challenging stage for neonatal mammals, as they are more susceptible to various stresses (i.e., oxidative stress) due to reduced maternal antibodies, changes in living conditions, and food transition from liquid to solid (17). The immune status of weaned puppies gradually weakens and become more susceptible to viral infections, such as canine distemper virus (CDV) and canine parvovirus (CPV). In dogs vaccinated against *Taenia hydatigena*, the combination of vitamin E and Se supplementation group dogs showed the best immune response (18). An optimal immune response to rabies primovaccination was observed in cattle supplemental Se with a dose of 3.6 mg/d, whereas, the 5.4 and 6.4 mg/d doses were only just above control levels, suggesting that over-supplementation could cause immunosuppression (19). Therefore, the effect of Se is closely related to the dosage and form of supplemental Se. Selenohomolanthionine [4,4′-selenobis (2-aminobutanoic acid), SeHLan] is a kind of organic Se that is biosynthesized in *Candida utilis*, whose metabolic pathway is simple and can synthesize selenoprotein more effectively than selenomethionine (SeMet) (20, 21). However, as a new Se source, researches of SeHLan on companion animals are rarely reported. In this study, the effects of supplementing different levels of SeHLan on antioxidant activity and immune efficacy in beagle puppies were investigated to provide a basis for the application of SeHLan.

## Materials and Methods

### Materials

Thirty 7-week-old male beagles with a weight of 3.05 ± 0.13 kg was purchased from Changzhou Bayle Experimental Animal Breeding Co., LTD [Ethics approval number SCXK (SU) 2018-0007]. Before the start of the experiment, all puppies underwent a normal basic physical examination and deworming, and the CPV antigen detection result was negative. Vanguard Plus 5 (Zoetis Inc., Lincoln, USA) was used for the vaccination of experimental puppies. Sodium selenite (SS) was purchased from Sigma and SeHLan was obtained from ABNA Trading Co., LTD. CPV strain was a generous gift from Dr. Wenbo Liu (Yangzhou University) and used for CPV antibody titers determination.

### Experimental Design and Animal Trial

In a completely randomized experimental design, 30 beagles were randomly divided into six groups of 5 each: the control group (–Se/–Vacc), immunization group (–Se/+Vacc), SS group (SS/+Vacc) and three SeHLan supplemented group (SeHLan-L/+Vacc, SeHLan-M/+Vacc, SeHLan-H/+Vacc). The control and immunization group feeds were not supplemented with Se, while the SS group received SS in 0.35 mg/kg of food dry matter (DM). The SeHLan groups were fed on diets supplemented with different doses of SeHLan (0.35, 1.0, 2.0 mg/kg of food DM). Puppies except the control group were vaccinated with Vanguard Plus 5, the control group puppies were inoculated with equal amount of saline. The first day of vaccination was recorded as day 0, a second vaccination was performed on day 21 post-immunization (PI) as a booster dose. SS and SeHLan diet were given to puppies on the first day of vaccination and lasted for 42 days.

According to the Guideline*s* for The Nutrition of Pet Food for Cats and Dogs issued by the European Pet Food Industry Federation (FEDIAF), the standard of nutrition for the healthy growth of dogs requires that each beagle's daily energy intake is not <397.5 kJ/kg BW (22, 23). The basic diet formula and nutrient composition (Table 1) were analyzed by Zhejiang Guozheng Inspection Technology Co., LTD. The SeHLan used in this experiment was a commercial Se product with a Se concentration of 4 g/kg, thus, the supplemented dose in each SeHLan group was calculated as Se. The right dose of SS and SeHLan powder for each puppy were thoroughly mixed with their own diet. All puppies were allowed *ad libitum* access to fresh water. The beagles were placed in unit cages in the isolation area of the experimental base of Zhejiang A&F University. The indoor temperature was maintained at 20- 25°C, and all experimental protocols were approved by the Animal Care and Use Committee of Zhejiang A&F University.

**Table 1:** Basic diet composition and nutrient level (Dry matter basis).

**Ingredients**	**%**	**Nutrition level**	**Contents**
Corn	28	Metabolic energy (MJ/kg)	16.45
Broken rice	12	Dry matter (g/100 g)	91.7
Wheat wheat	12	Crude fat crude fat (%)	15.2
Fishmeal	5	Calcium (%)	0.96
Chicken powder	25	Crude fiber crude fiber (%)	1.4
Egg powder	6	Crude protein (%)	27.1
Grease	4	Methionine (g/100 g)	0.45
Puree	6	Lysine lysine (g/100 g)	1.39
Dried carrots	2	Selenium (mg/kg)	0.06
Total	100	Total phosphorus total (%)	0.69

### Sample Collection and Preparation

Blood samples were collected from the canine cephalic vein on 0, 7, 14, 21, 28, 35, and 42 d PI. Part of the whole blood anticoagulated with heparin was separated and used for peripheral blood mononuclear cells (PBMCs) proliferation test and polymorphonuclear neutrophils (PMN) phagocytosis test. Besides, canine serum was collected and stored at −20°C for later use in the determination of serum Se concentration, glutathione peroxidase (GSH-Px), superoxide dismutase (SOD), malondialdehyde (MDA), cytokines (IL-2, IL-4), and CPV hemagglutination inhibition (HI) test.

### Determination of Serum Se Concentration

The serum Se concentration was determined according to the method described by Donadio et al. (24) using a hydride generation-atomic absorption spectrophotometry. The serum is rapidly digested with a mixture of nitric and perchloric acids at a temperature of 180 ± 10°C, and hydrochloric acid is used to reduce Se (VI) to Se (IV).

### Determination of the Activity of Serum Antioxidant Parameters

GSH-Px, SOD, and MDA levels were determined using assay kits obtained from Jiancheng Biochemical Co., LTD. (Nanjing, Jiangsu, China), according to the manufacturer's instructions. Briefly, GSH-Px activity was determined by the colorimetric method; SOD activity was determined by the hydroxylamine method (WST-1); MDA was assayed by the thiobarbituric acid (TBA) method. All experiments were performed in triplicate.

### ConA-Induced PBMCs Proliferation Assay

The isolation of PBMCs and PMNs from whole blood was performed by the gradient density method using Histopaque-1077 and 1119 (Sigma, USA) simultaneously. In brief, 2.5 ml of whole blood was mixed with equivalent volume D-Hank's and layered onto 2.5 ml Histopaque-1077 and 2.5 ml Histopaque-1119 in 15 ml tubes. After centrifugation at 2,000 r/min for 20 min, the mixed solution was divided into six layers: plasma layer, mononuclear cells layer, cell separation solution layer (1077), granulocyte layer, cell separation solution layer (1119) and red blood cell layer. PBMCs were washed twice in D-Hank's and re-suspended in 1 ml RPMI^+^ (RPMI-1640 with 100 Units/ml of Penicillin and 100 μg/ml of Streptomycin, 2 mM L-glutamine, and 10% heat-inactivated fetal calf serum), and stained with trypan blue to assess cell viability and ensure that the number of viable cells was above 90%. The cell suspension concentration was adjusted to 1 × 10^6^/ml, dispensed at 100 μl/well in a 96-well plate, and ConA (Sigma, USA) was added at a final concentration at 2.5 μg/ml in the sample groups. In the control group: 100 μl PBMCs suspension and 100 μl RPMI^+^, and in the Zero Group: 200 μl RPMI^+^ were added. All the plates were incubated for 48 h at 37°C and 5% CO_2_, and MTT (5 mg/mL) was added and incubated for 4 h. DMSO was used for lysis and dissolution of the crystals and the absorbance of each well was measured at OD_570_. The stimulation index (SI) was used to determine the PBMCs proliferation function, SI = (sample group OD_570_ Value – zero group OD_570_ Value)/(control group OD _570_ Value – zero group OD_570_ Value).

### Enzyme-Linked Immunosorbent Assay for Detection of IL-2 and IL-4

The serum levels of IL-2 and IL-4 were determined using sandwich ELISA kits obtained from Wuhan Huamei Biotech Co., LTD (Wuhan, Hubei, China) according to the manufacturer's instructions individually. Optical density (OD) was measured at 450 nm for IL-2 and IL-4 using an automated microplate reader (BioTek SynergyTM H1, USA). All samples were tested in triplicate reactions.

### Determination of PMN Phagocytosis of FITC-Labeled Bacteria

The isolated PMN from whole blood were washed twice in D-Hank's and re-suspended in 1 ml RPMI^+^ at a concentration of 0.5 × 10^6^/ml. *Staphylococcus aureus* (*S. aureus*) was cultured overnight and inactivated at 80°C for 1 h. Bacterial were washed and re-suspended in 1 ml PBS and labeled with 5 mg/ml fluorescein isothiocyanate (FITC, Sigma, USA) in 100 μl by incubating at room temperature (RT) for 1.5 h. The FITC bacteria were washed and re-suspended to 1 × 10^9^/ml in PBS and stored at 4°C in the dark for subsequently use. 500 μl PMN suspension and 25 μl FITC-labeled *S. aureus* were thoroughly mixed and incubated at 28°C for 10 min. The mixed samples were centrifuged at 300 r/min for 5 min at 4°C, and the sediments were washed twice with D-Hank's solution and fixed with 1% paraformaldehyde. The percentage phagocytic activity of PMN was analyzed by flow cytometry (BD Accuri C6, USA). Generally, 10,000 cells were measured for each sample.

### CPV HI Test

CPV antibodies were assayed as described by Carmichael et al. (25). Briefly, porcine whole blood was diluted 1:10 (v/v) with Alsever's solution and stored at 4°C. Before use, the solution was centrifuged at 1,500 r/min for 5 min and the supernatant was discarded. The sediments were washed three times with phosphate buffered solution (PBS, pH 7.2), then 1% porcine erythrocytes suspension was prepared in PBS. The CPV strain titer was determined by hemagglutination (HA) assay. A two-fold serial dilution of the virus was added in a V-96-well plate, mixed with equal volume 1% erythrocytes, and then incubated at 4°C for 1 h. The hemagglutination unit (HAU) was defined as the highest dilution that completely agglutinated reciprocal, and four HAU was used for HI experiments. The serum samples with double gradient dilution and 4 HAU of CPV were mixed and incubated at RT for 30 min, and then 1% erythrocytes were added and incubated at 4°C for 1 h. The CPV HI titers of the serum were determined based on the highest dilution of the four HAU that were completely inhibited and presented as log base 2 in the figure Y-axis. The protective antibody titer (PAT) of CPV was ≥1:80 (26), and log base 2 of 80 was 2^6.3^. Each serum sample was tested in triplicate.

### Statistical Analysis

All statistical analyses were performed with SPSS Statistics 21.0 (IBM, USA). One-way ANOVA was used to compare the differences between groups. All values in each treatment group are presented as mean ± standard deviation. *P* < 0.05 was considered significant.

## Results

### SeHLan Significantly Increased Serum Se Concentration in Weaned Puppies

**Figure 1:**
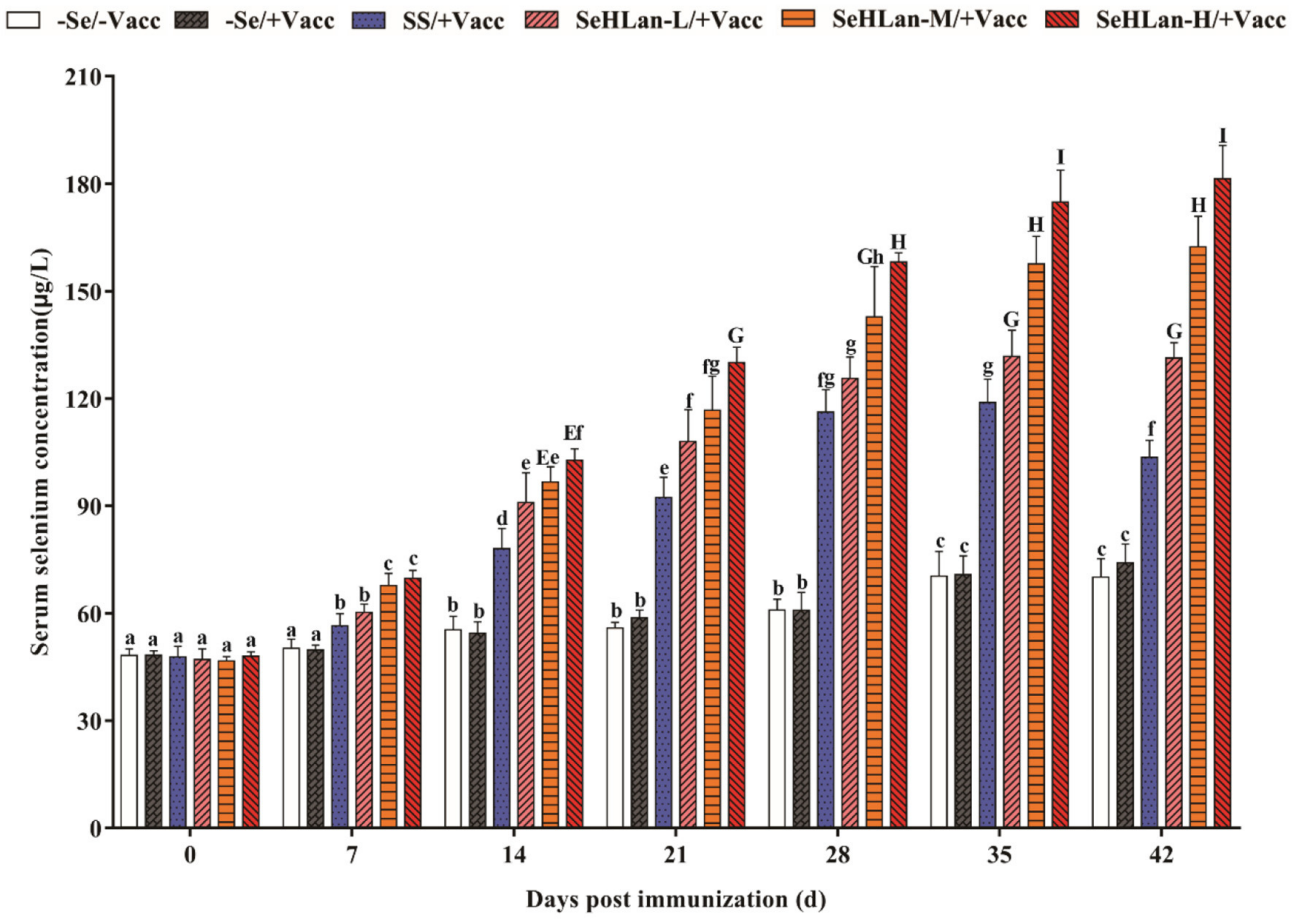
Effects of Se supplementation on serum Se concentration of puppies at different days post-immunization. All values in each treatment group are presented as means ± SD (*n* = 5). Within a panel, bars labeled with the same letters but different capitalization indicate significant differences (*P* < 0.05), and the absence of the same letters indicate extremely significant differences (*P* < 0.01). –Se/–Vacc: puppies were fed with basal diet and unvaccinated; –Se/+Vacc: puppies were fed with basal diet and vaccinated with Vanguard Plus 5; SS/+Vacc: puppies were fed with SS (0.35 mg/kg DM) and vaccinated; SeHLan-L(M/H)/+Vacc: puppies were fed with SeHLan (0.35, 1, 2 mg/kg DM, respectively) and vaccinated.

As shown in Figure 1, there was no significant difference in serum Se concentration between the –Se/–Vacc and –Se /+Vacc groups during the entire experiment. The serum Se concentration in the two Se unsupplementation groups showed a significantly increase on 14 d PI, and a 1.45-fold increase in serum Se concentration was observed in the control group on 42 d PI compared with that on 0 d PI. While the serum Se concentration in the SS and SeHLan groups showed an extremely high increase on 7 d PI (*P* < 0.01). The serum Se concentration of puppies in the SeHLan-L/+Vacc group was significantly higher compared with that in the SS group during 14~42 days PI (*P* < 0.05). Besides, the serum Se concentration gradually increased in the SeHLan groups with an increase in supplementation. The most significant increase of serum Se concentration of puppies was observed in the SeHLan-H/+Vacc group with a 3.78-fold increase on 42 d PI compared with that on 0 d PI.

### SeHLan Significantly Improved Serum Antioxidant Activity in Weaned Puppies

**Figure 2:**
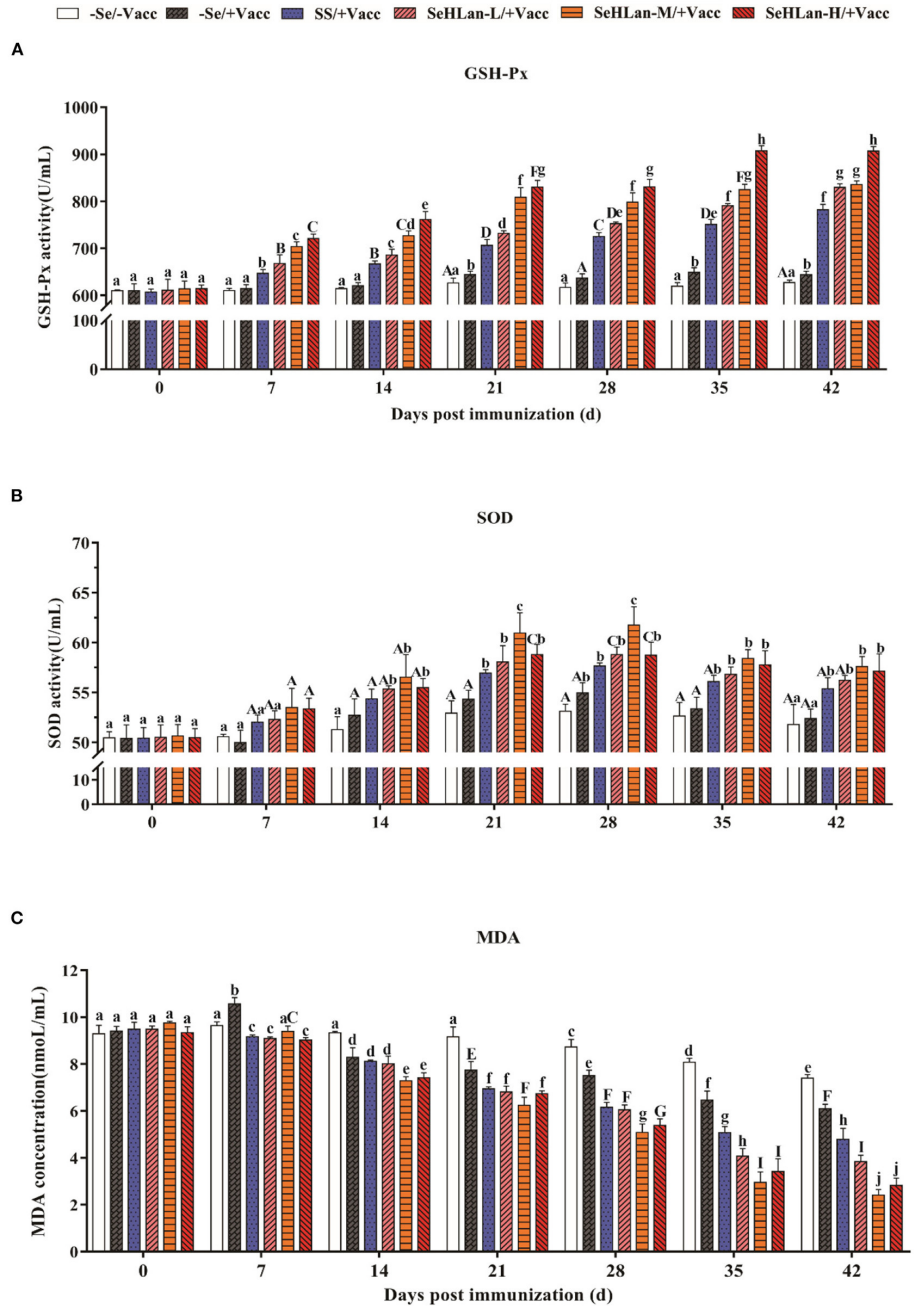
The influence of Se Supplementation on serum GSH-Px **(A)**, SOD **(B)** activities and MDA **(C)** content of puppies at different days post-immunization. All values in each treatment group are presented as means ± SD (*n* = 5). Within a panel, bars labeled with the same letters but different capitalization indicate significant differences (*P* < 0.05), and the absence of the same letters indicate extremely significant differences (*P* < 0.01). –Se/–Vacc: puppies were fed with basal diet and unvaccinated; –Se/+Vacc: puppies were fed with basal diet and vaccinated with Vanguard Plus 5; SS/+Vacc: puppies were fed with SS (0.35 mg/kg DM) and vaccinated; SeHLan-L(M/H)/+Vacc: puppies were fed with SeHLan (0.35, 1, 2 mg/kg DM, respectively) and vaccinated.

An upward trend of serum GSH-Px activity in puppies were observed in the control and immunization groups throughout the study (Figure 2A). The serum GSH-Px activity in the SS and SeHLan supplementation groups significantly increased on 7 d PI. SS showed lower GSH-Px activity compared with the SeHLan supplementation group with a same Se dosage (0.35 mg/kg DM). The serum GSH-Px activity significantly increased with an increase in the dosage of SeHLan (*P* < 0.01), which was similar to the changes in serum Se concentration.

The activity of serum SOD in all groups with or without Se supplementation increased and peaked around 28 d PI, then decreased gradually (Figure 2B). The increased range of serum SOD activity was significantly higher in SS and the three SeHLan supplementation groups compared with the control group. There was no significant difference in serum SOD activity between the SS/+Vacc and SeHLan-L/+Vacc groups. Notably, the serum SOD activity in the SeHLan-M/+Vacc group was highest and showed a significant difference on 28 d PI.

The serum MDA content remained stable for 21 days in the control group at the beginning of the experiment and later began to decline (Figure 2C). Serum MDA levels were significantly decreased in the SS group and the three SeHLan supplementation groups on 7 d PI (*P* < 0.01). Furthermore, the decrease was significantly higher in all Se supplementation groups than in the control group and the immunization group throughout the study (*P* < 0.01). Among all the groups, the serum MDA content decreased the most in the moderate SeHLan supplementation group (1.0 mg/kg DM).

### SeHLan Significantly Improved the Proliferation of PBMCs in Weaned Puppies

**Figure 3:**
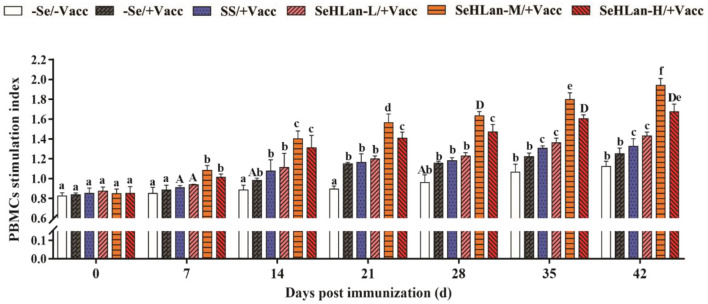
The influence of Se supplementation on PBMCs stimulation index of puppies at different days post-immunization. All values in each treatment group are presented as means ± SD (*n* = 5). Within a panel, bars labeled with the same letters but different capitalization indicate significant differences (*P* < 0.05), and the absence of the same letters indicate extremely significant differences (*P* < 0.01). –Se/–Vacc: puppies were fed with basal diet and unvaccinated; –Se/+Vacc: puppies were fed with basal diet and vaccinated with Vanguard Plus 5; SS/+Vacc: puppies were fed with SS (0.35 mg/kg DM) and vaccinated; SeHLan-L(M/H)/+Vacc: puppies were fed with SeHLan (0.35, 1, 2, respectively) and vaccinated.

The proliferation of PBMCs showed no significant changes in the first 21 d in the experiment but later gently increased in the control group (Figure 3). Vaccination stimulated the proliferation of PBMCs which was significantly higher in the immunization group compared with the control group between 14~28 days PI (*P* < 0.05). A significant increase in PBMCs proliferation was observed in SS and SeHLan supplementation groups. Besides, the increase in PBMCs proliferation was most significant in the SeHLan-M/+Vacc group compared with the control group on 42 d PI (*P* < 0.01).

### SeHLan Significantly Increased Serum IL-2 and IL-4 Concentration in Weaned Puppies

**Figure 4:**
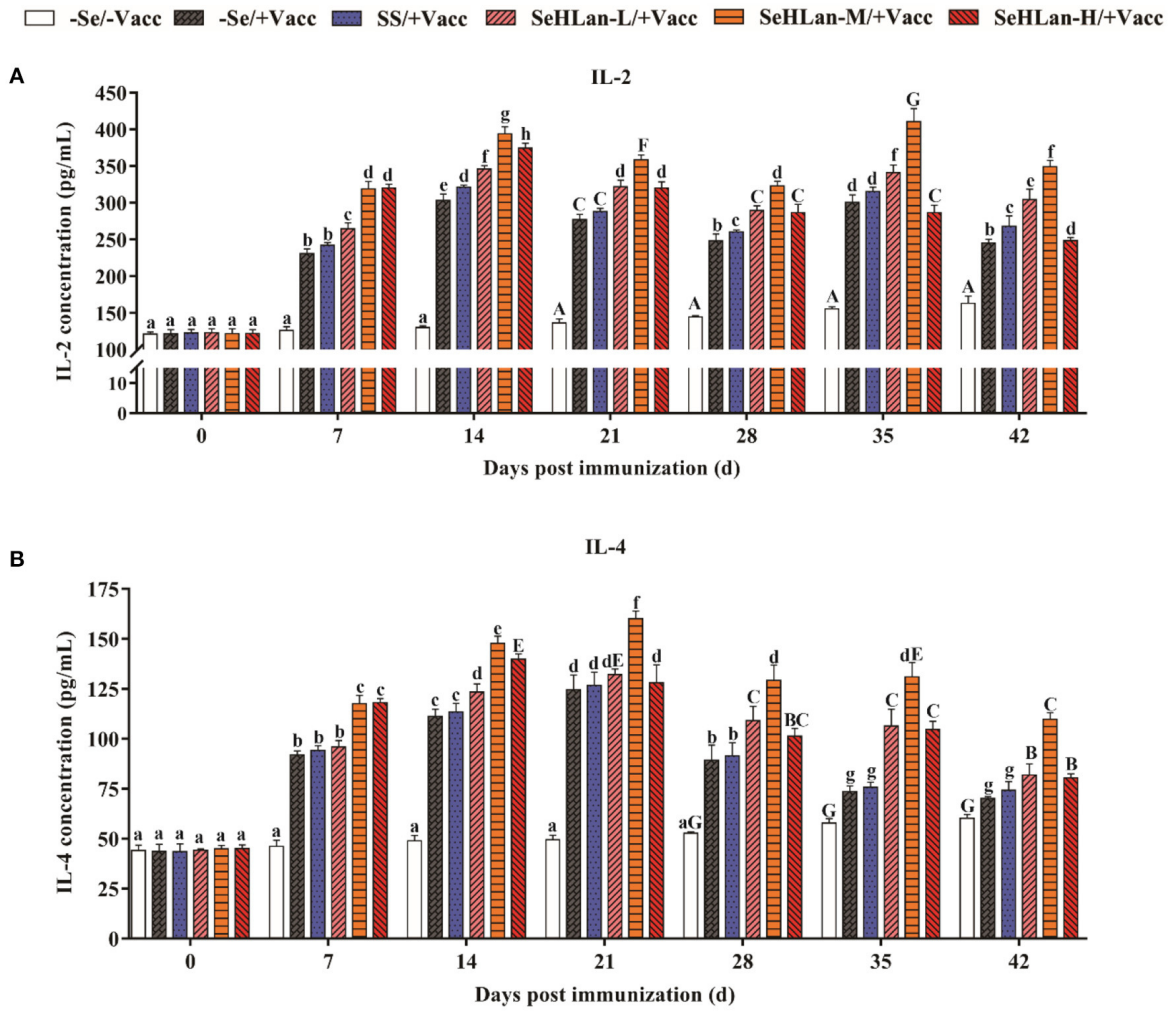
The influence of Se supplementation on serum IL-2 **(A)** and IL-4 **(B)** concentration of puppies at different days post immunization. All values in each treatment group are presented as means ± SD (*n* = 5). Within a panel, bars labeled with the same letters but different capitalization indicate significant differences (*P* < 0.05), and the absence of the same letters indicate extremely significant differences (*P* < 0.01). –Se/–Vacc: puppies were fed with basal diet and unvaccinated; –Se/+Vacc: puppies were fed with basal diet and vaccinated with Vanguard Plus 5; SS/+Vacc: puppies were fed with SS (0.35 mg/kg DM) and vaccinated; SeHLan-L(M/H)/+Vacc: puppies were fed with SeHLan (0.35, 1, 2 mg/kg DM, respectively) and vaccinated.

No significant changes were reported both in serum of IL-2 and IL-4 concentration in the first 14 d in the experiment but later gently increased in the control group (Figure 4). However, a two-fold increase both in IL-2 and IL-4 production was observed in the immunization group compared with the control group on 7 d PI. Se supplementation contributed to IL-2 and IL-4 production. The improved effect of serum IL-2 and IL-4 concentration at low dosage SeHLan supplementation (0.35 mg/kg DM) was higher than that in the SS supplementation group (*P* < 0.05). Among the three SeHLan supplementation groups, the SeHLan-M/+Vacc group showed the greatest improvement in serum concentration of IL-2 on 35 d PI and IL-4 on 21 d PI, respectively. The serum IL-2 concentration peaked two times on 14 d and 35 d PI, respectively. However, serum IL-4 concentration peaked on 21 d PI in the SeHLan-M/+Vacc group, then decreased gradually, and showed significant difference compared with the immunization group.

### SeHLan Significantly Improved PMN Phagocytosis in Weaned Puppies

**Figure 5:**
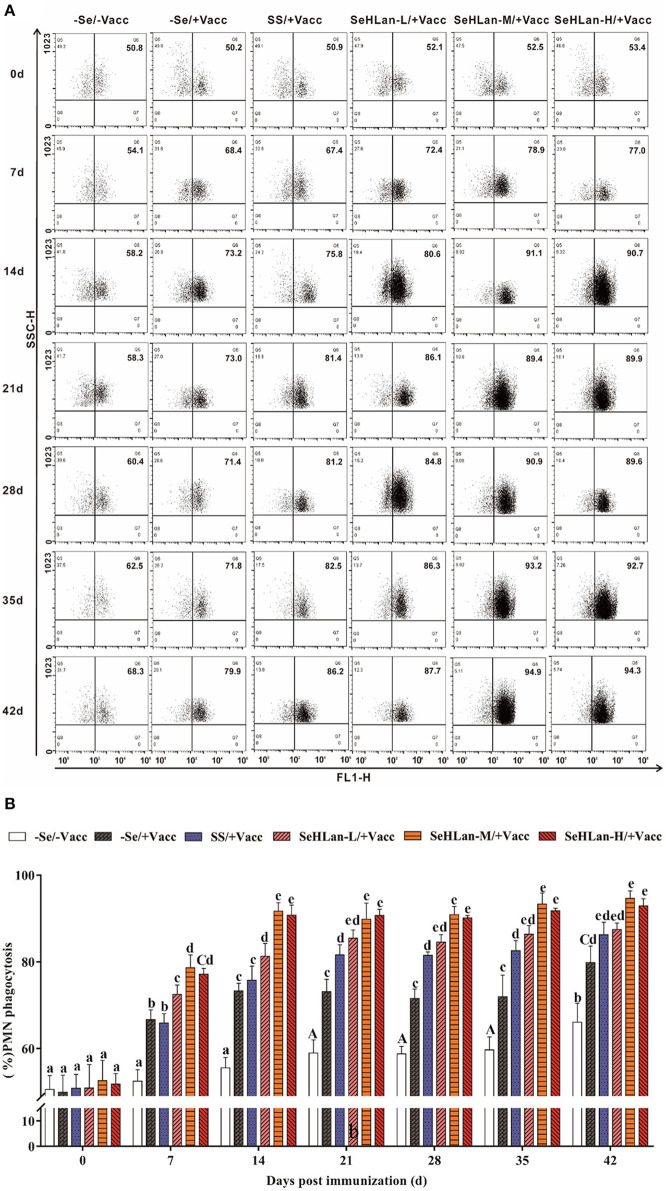
The influence of Se supplementation on PMN phagocytosis of puppies at different days post-immunization. **(A)** Flow cytometric analysis of the phagocytosis of the *S. aureus* treated PMN. **(B)** Quantitative analysis of PMN undergoing phagocytosis. All values in each treatment group are presented as means ± SD (*n* = 5). Within a panel, bars labeled with the same letters but different capitalization indicate significant differences (*P* < 0.05), and the absence of the same letters indicate extremely significant differences (*P* < 0.01). –Se/–Vacc: puppies were fed with basal diet and unvaccinated; –Se/+Vacc: puppies were fed with basal diet and vaccinated with Vanguard Plus 5; SS/+Vacc: puppies were fed with SS (0.35 mg/kg DM) and vaccinated; SeHLan-L(M/H)/+Vacc: puppies were fed with SeHLan (0.35, 1, 2 mg/kg DM, respectively) and vaccinated.

As shown in Figures 5A,B, there was no significant difference in PMN phagocytosis in the control group until on 21 d PI. However, the immunization group showed a sharp increase in PMN phagocytosis following vaccination. Se supplementation increased the phagocytic ability of PMN. There was no significant difference in PMN phagocytosis between the –Se/+Vacc and SS/+Vacc groups in the first 14 d (*P* > 0.05), however, during the trial period, PMN phagocytosis in the SS/+Vacc group was significantly higher compared with that in the –Se/+Vacc group (*P* < 0.01). Among all the Se supplementation groups, the improved effect of PMN phagocytosis in the SeHLan group was higher than that in the SS group. The SeHLan-M/+Vacc and SeHLan-H/+Vacc groups showed the similar and higher phagocytic ability of PMN, with the phagocytic rate maintained above 90% between 14 ~ 42 d PI, and was significantly higher than that in the SeHLan-L/+Vacc group (*P* < 0.05).

### SeHLan Significantly Improved CPV HI Titers in Weaned Puppies

**Figure 6:**
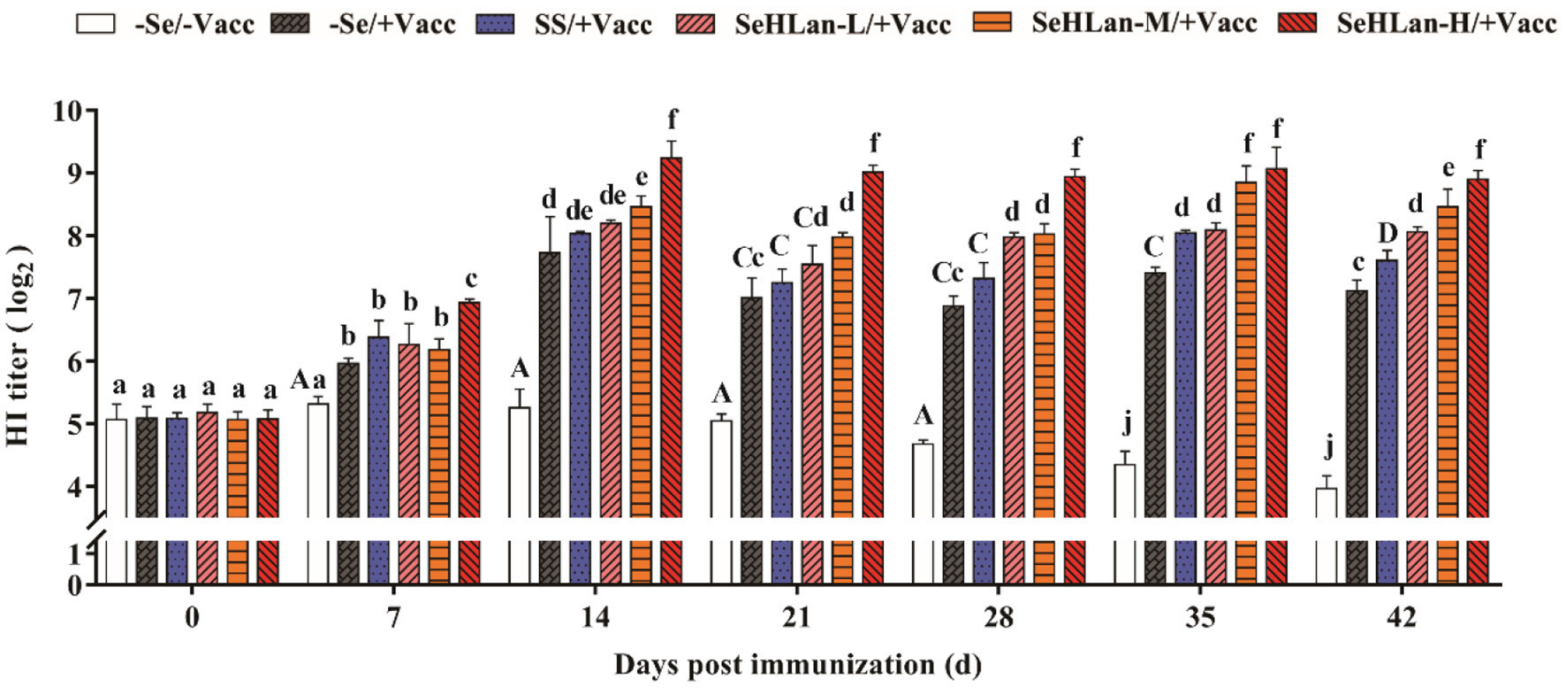
Effect of Se supplementation on serum CPV HI titers of puppies at different days post-immunization. All values in each treatment group are presented as means ± SD (*n* = 5). Within a panel, bars labeled with the same letters but different capitalization indicate significant differences (*P* < 0.05), and the absence of the same letters indicate extremely significant differences (*P* < 0.01). –Se/–Vacc: puppies were fed with basal diet and unvaccinated; –Se/+Vacc: puppies were fed with basal diet and vaccinated with Vanguard Plus 5; SS/+Vacc: puppies were fed with SS (0.35 mg/kg DM) and vaccinated; SeHLan-L(M/H)/+Vacc: puppies were fed with SeHLan (0.35, 1, 2 mg/kg DM, respectively) and vaccinated.

The CPV antibody titers were similar and below the PAT of 2^6.3^ at the start of the experiment in all puppies. However, during the experiment period, the CPV HI titers increased and then decreased, with a small range variation and still under PAT levels. As shown in Figure 6, CPV HI titers increased in all the vaccinated puppies but remained below the PAT level on 7 d PI, except in the SeHLan-H/+Vacc group. A rapid increase in CPV HI titers was observed on 14 d PI in all vaccinated puppies, and all the titers were higher than the PAT level. The results showed that Se supplementation improved CPV antibody production. With a same Se dosage, the promoting effects of CPV antibody showed a significant difference between the SeHLan-L/+Vacc (0.35 mg/kg DM) group and the SS group (*P* < 0.05). Among the three SeHLan supplementation groups, the SeHLan-H/+Vacc group showed the highest promoting effect for CPV antibody titers.

## Discussion

In this study, we investigated the antioxidative and immunomodulatory effects of SeHLan in weaned puppies during a vaccination period. Serum Se concentration is sensitive to changes in diet supplemented with extra Se source and can be used as a biomarker of Se concentration in dogs (22, 23). In this study, the daily intake of the basal diet was increased based on the age and weight of puppies in the control and immunization groups. The Se concentration of the basal diet was 0.06 mg/kg, hence, the serum Se concentration in the two groups was moderately increased. According to AAFCO, the minimum and maximum amount of Se used in dog food are 0.35 and 2.0 mg/kg DM, meaning that the dosage of SS and SeHLan adopted in this study was safe. The results showed that there was a dose-dependent relationship between serum Se concentration and the amount of SeHLan in all the three SeHLan supplementation groups. Moreover, the serum Se concentration in the SeHLan supplementation groups was significantly higher than in the SS group supplied with equivalent doses. Similar results were observed in lambs treated with different sources of Se (27).

The level of immunity in puppies at weaning is relatively low and they are susceptible to a variety of diseases. Similarly, the frequency of infections in puppies with diarrhea around weaning is high. A recent study reported that selenium-enriched yeast elevated the GSH-Px levels and decreased the MDA content in laying ducks (28). As expected, the antioxidative capacity was significantly increased by Se in this study, and the promoting effects of SeHLan supplementation were better than that in SS supplied with equivalent doses. As a Se containing peroxidase, GSH-Px is a major group of enzymes that eliminate hydrogen peroxide which is extremely harmful to the cell. In the present study, SeHLan promoted the GSH-Px activity in a time and dose-dependent manner. The moderate doses of SeHLan supplementation (1 mg/kg DM) exhibited the highest increase in SOD activity and the highest decrease in MDA content in serum. However, significant changes in the antioxidative capacity were observed in the control and immunization groups, which was largely attributed to the increase in age. Similarly, SeMet supplementation in the diet significantly improved the antioxidant capacity and plasma Se concentration in weaning piglets (29). Moreover, oxidative stress-induced intestinal mucosa disruption were attenuated by adding selenium-enriched yeast in weaned pigs in a HO-1/Nrf2 pathway, which is a critical transcription signal in antioxidant enzymes production (17).

PMN constitute the first line of defense in the protection of the host from invading microorganisms. During phagocytosis, ROS are produced and the consequent production of free radicals which are eliminated through the antioxidant defense system (30). Administration of Se to goats fed on Se-deficient diet resulted in increased PMN function which was associated with physiologic changes in the GSH-Px level (31). As expected, the phagocytic ability of PMN increased in the SS and three SeHLan supplementation groups in this study. Numerous researches have confirmed that Se enhances the function of various kinds of immunocompetent cells. PBMCs proliferation is routinely used to evaluate lymphocyte response stimulated by non-specific mitogens. In the present study, vaccination with Vanguard Plus 5 increased PBMCs proliferation and PMN phagocytosis and promoted the production of IL-2 and IL-4. Meanwhile, Se supplementation in basal diet improved the immune response, while the moderate doses of SeHLan supplementation (1 mg/kg DM) showed the greatest-promoting effect. IL-2 is mainly produced by type 1 T helper (Th1) cells and plays a significant role in macrophage activation and in promoting a cell-mediated immune response (32). Previous studies have reported consistent results, that Se promotes glutathione peroxidase (GPx1) and thioredoxin reductase 1 (TR1) expression, and enhances ConA induced T-cell activation and secretion of IL-2 in porcine splenocytes (33). The ability of Se to induce augmented expression of IL-2 appears to occur through the increased expression of the IL-2 receptor (34). As a major product of type 2 T helper (Th2) cells, IL-4 is also a potent inducer of Th2 differentiation (35). Besides, IL-4 can promote B lymphocyte proliferation and immunoglobulin secretion (36). In this study, maternal antibody serum HI titers declined to 2^5^ at 7 weeks of age and kept decreasing in the control group, and the levels were below the PAT level. About 14 days were needed after vaccination to achieve a protective titer against CPV. Therefore, the lack of a protective stage was highly important for puppies needing more protection. In a previous study, Se supplemented chicken showed higher IgM and IgY titers after vaccination with low pathogenicity avian influenza virus vaccine (37). Besides, a previous study in humans showed that the mRNA expression levels of selenoprotein S (SEPS1) significantly increased 7 days after an influenza vaccine challenge with Se supplementation (38). As shown in our study, Se supplementation not only improved the CPV antibody HI titers but also shortened antibody production time. The high doses of SeHLan supplementation (2 mg/kg DM) manifested the greatest CPV HI titers promoting effect.

## Conclusions

The present study suggested that dietary SeHLan supplementation improved the antioxidant activity, increased CPV antibody HI titers, and enhanced the immune function in puppies after weaning. Moreover, the beneficial effects of SeHLan were superior to those in the SS group supplied with equivalent doses of SS. Finally, we conclude that the right amount of SeHLan (1~2 mg/kg DM) can serve as a potential nutrition additive in puppy feeding.

## Data Availability Statement

The original contributions presented in the study are included in the article/supplementary material, further inquiries can be directed to the corresponding author/s.

## Ethics Statement

This study was approved by the Institutional Animal Care and Use Committee of Zhejiang Province (Permit Number: SYXK-2018-0010).

## Author Contributions

XW and HS conceived and designed the study. CS, MZ, ZY, and TM performed the experiments. SJ, BZ, and QS analyzed the data. CS wrote the manuscript. YZ, WD, DL, and YG revised it critically for important content. All authors have read and approved the final version of the manuscript.

## Funding

This study was supported by Zhejiang Provincial Natural Science Foundation (Nos. LQ19C180003 and LQ20C180002); the National Natural Science Foundation of China (Nos. 31902249, 31802258, and 31602119); Zhejiang A&F University (Nos. 2015FR042, 2018FR009, 2018FR015, and 2019FR013); Department of Education of Zhejiang Province (No. Y202044917); and Key Research and Development Program of Zhejiang Province (2019C02043).

## Conflict of Interest

DL and YG were employed by ABNA Trading (Shanghai) Co., Ltd. The remaining authors declare that the research was conducted in the absence of any commercial or financial relationships that could be construed as a potential conflict of interest.

## Publisher's Note

All claims expressed in this article are solely those of the authors and do not necessarily represent those of their affiliated organizations, or those of the publisher, the editors and the reviewers. Any product that may be evaluated in this article, or claim that may be made by its manufacturer, is not guaranteed or endorsed by the publisher.
